# The complexity of cortical folding is reduced in chronic cocaine users

**DOI:** 10.1111/adb.13268

**Published:** 2023-02-01

**Authors:** Nicolò Trevisan, Fabio Di Camillo, Niccolò Ghiotto, Giulia Cattarinussi, Maddalena Sala, Fabio Sambataro

**Affiliations:** ^1^ Department of Neuroscience (DNS) University of Padova Padua Italy; ^2^ Padova Neuroscience Center University of Padova Padua Italy; ^3^ Present address: Department of Pathophysiology and Transplantation University of Milan Milan Italy

**Keywords:** addiction, cocaine, complexity of cortical folding, fractal dimension, grey matter

## Abstract

Cocaine use is a worldwide health problem with psychiatric, somatic and socioeconomic complications, being the second most widely used illicit drug in the world. Despite several structural neuroimaging studies, the alterations in cortical morphology associated with cocaine use and addiction are still poorly understood. In this study, we compared the complexity of cortical folding (CCF), a measure that aims to summarize the convoluted structure of the cortex between patients with cocaine addiction (*n* = 52) and controls (*n* = 36), and correlated it with characteristics of addiction and impulsivity. We found that patients with cocaine addiction had greater impulsivity and showed reduced CCF in a cluster that encompassed the left insula and the supramarginal gyrus (SMG) and in one in the left medial orbitofrontal cortex. Finally, the CCF in the left medial orbitofrontal cortex was correlated with the age of onset of cocaine addiction and with attentional impulsivity. Overall, our findings suggest that chronic cocaine use is associated with changes in the cortical surface in the fronto‐parieto‐limbic regions that underlie emotional regulation and these changes are associated with earlier cocaine use. Future longitudinal studies are warranted to unravel the association of these changes with the diathesis for the disorder and with the chronic use of this substance.

## Graphical abstract

Chronic cocaine use is reduced with altered complexity of cortical folding in the insula and in the orbitofrontal cortex. Reduced cortical complexity correlated with the severity of the addiction and with impulsivity. Cortical complexity changes may be associated with the risk of developing addiction as well as the reinforcement of the use of the substance itself.

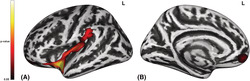

## 1 INTRODUCTION

According to the 2021 yearly report of the Office of Drug and Crime of the United Nations, around 275 million people have used drugs, approximately 5.4% of the global population aged 15‐64 years, thus increasing by 22% from 2010 and forecasting a further increase by 11% by 2030.^1^ Only in the United States over 70 000 drug overdoses occur annually, of which 21.2% are due to cocaine.^1^ Cocaine‐associated deaths also include long‐term organic disability and neurocognitive deficits in attention, executive function, verbal memory and processing speed that could derive from sequelae of cardiovascular events due to the effects of this substance.^2^ Moreover, the co‐occurrence of psychiatric conditions, including generalized anxiety disorder, depression, attention deficit hyperactivity disorder (ADHD) or conduct disorder, is commonly observed.^3^

Recent meta‐analytic evidence has shown that patients with cocaine addiction (CA) present changes in brain structure with significantly lower grey matter (GM) volume in the right superior temporal gyrus, right insula and right postcentral gyrus compared with healthy controls (HCs), as well as increased GM volume in the right inferior parietal gyrus.^4^ In cocaine users, lower GM volume has been reported in the prefrontal and temporal cortex, the insula, the striatum and the thalamus.^5^ Interestingly, GM alterations show a relationship with the characteristics of substance use. Indeed, the duration of drug intake has been associated with abnormal GM volume in the right insula, the right gyrus rectus, the bilateral middle temporal gyrus and the right inferior frontal gyrus,^6^ whereas trait and behavioural impulsivity have been associated with the reduction of GM volume in fronto‐parietal areas in cocaine users.^7^

Although brain volume is related to surfaced‐base measures, these latter indexes may be more sensitive to cortical reductions, as shown in several neuropsychiatry disorders and aging.^8^ As expected, alterations in cortical measures have been shown in cocaine users. Particularly, in non‐treatment‐seeker cocaine users, Geng and colleagues^9^ observed a reduction in cortical thickness (CT) in the bilateral insula and an increase in the bilateral temporal lobe, which are crucial areas for the integration of visceral sensations, which can affect the decision‐making process in addiction.^10^ In addition, decreased CT was described in the superior frontal gyrus, inferior frontal gyrus and orbitofrontal cortex (OFC) in cocaine users, as well as smaller cortical surface area in the anterior cingulate cortex (ACC).^11^ Conversely, a recent investigation exploring gyrification in the OFC in cocaine users reported no significant differences from the control group.^11^

Recently, novel measures have been introduced to describe the morphology of the cortical surface and to assess the cortical complexity in the fractal dimension (FD). FD is a non‐linear measure derived from fractal geometry that summarizes morphological aspects of an object by providing a numerical value of self‐similarity, as a way to outperform traditional Euclidian geometry for the description of complex structures.^12^ In this sense, FD can be defined as a complexity index that assesses how a detail in a fractal pattern varies across multiple measuring scales. Considering that the highly convoluted brain cortex represents a fractal structure, we can apply this concept to the study of GM, as its complexity of cortical folding (CCF) can be examined through fractal geometry tools.^12^ FD can be used to describe cortical complexity in healthy and clinical populations, as it is sensitive to detect morphological changes related to pathological and developmental changes.^13^ Compared to voxel‐based morphometry (VBM), surface‐based analyses appear to be less susceptible to inaccuracies of anatomical normalization during preprocessing, likely contributing to the heterogeneity of volumetric results.^14^ CCF can provide quantitative information on cortex convolution, gathering CT, sulcal depth and folding area in a single numeric value.^15^ CCF is more temporally stable than volume‐based measures of GM.^16^ Moreover, because the CCF summarizes distinct but linked elements of GM surface in a single measure, including CT, sulcal depth and surface area, it could result in greater sensitivity in detecting changes in the brain relative to each index.^12^ This measure increases from fetal age to adulthood, until it starts a slow and stable decrease until later in life.^17^ CCF changes have been found in several neurological and psychiatric disorders, including multiple sclerosis, dementia, stroke and schizophrenia,^18^ and most studies show a reduction in CCF, thus suggesting that this measure may reflect alterations in brain function, for example, in multiple sclerosis, in which it predicts the worsening of disability.^19^ Interestingly, findings from human studies and animal models examined how impulsivity, a construct commonly defined as deficit in the behaviour inhibition, is a risk factor for the emergence of substance use disorders (SUDs).^20^ In general, deficits in impulse control have been consistently reported in subjects with SUD. Addiction is usually associated with an impairment in the ability to ignore drug‐related stimuli, but attentional biases in patients with SUD are also present in more general nonspecific reward‐related situations.^21^ Attentional biases could be one of the mechanisms by which impulsivity affects addictive behaviours. This may be caused by a bias of classical conditioning processing and by effects on the dopaminergic system.^22^ Several instruments have been developed to measure the different facets of impulsivity, including the Barratt Impulsivity Scale (BIS‐11), which allows the evaluation of a specific subdomain of attentional impulsiveness.^23^ Structural MRI studies have been carried out to determine the neural correlates of impulsivity, both in HCs and in clinical populations with substantial impulsivity, including patients with ADHD and bipolar disorders. In the healthy population, the volume of the OFC has been shown to correlate negatively with the impulsivity measured by the BIS scale.^24^ Specifically, attentional impulsivity was negatively correlated with temporal gyrus volume in HCs, whereas OFC was negatively associated in patient with impulsive behaviour.^25^

In this study, our objective was to investigate CCF in patients with CA using FD. Moreover, impulsivity was generally associated with cocaine use and the development of its addiction, with patient with CA usually showing higher scores on the BIS‐11 scale compared with HCs.^26^ In particular, high impulsivity also predicts the shift from impulsivity to compulsivity during the development of addictive behaviours.^27^ In agreement with this, neuroimaging studies have shown a relationship between impulsivity and cortical volume and the surface area of the frontal, temporal and insular cortex.^26^ Because these regions have been shown to play an important role not only in impulsivity but also in decision‐making,^7^ we hypothesized that CCF in the brain of patients with CA would be altered in these regions. Finally, we predicted an association between CCF changes and (1) the duration of cocaine use due to its widespread effects on brain structure and (2) impulsivity (in particular, for the attentive subdomain) in the regions implicated in the predisposition to addiction. To reduce the heterogeneity of neural effects due to sex differences,^28^ we limited our investigation to men.

## 2 MATERIAL AND METHODS

### 2.1 Subjects

CA and HC were selected from the Mexican database on cocaine use disorder.^29^ This open database contains demographic, clinical and imaging data from 145 subjects who were recruited as part of a project on the study of addictions. CA was assessed using the MINI Mini‐PLUS interview in Spanish version 5.0.0 that uses the diagnostic and statistical manual of mental disorders (DSM‐IV) criteria. Additionally, the instant view drug screening test was applied to screen for illicit substances other than cocaine (amphetamines, methamphetamines, benzodiazepines, cannabis and opioids), thus excluding participants who showed a current dependence (based on the DSM‐IV criteria) of substances other than cocaine and nicotine. Moreover, lifetime use of other drugs was evaluated using the Addiction Severity Index. Due to the low number of female participants in the study and to reduce sex‐dependent heterogeneity,^28^ we selected only male participants. To evaluate the association between CCF and cocaine use characteristics, we excluded participants from the CA group whose daily cocaine intake was not specified in the database. Furthermore, participants with a history of schizophrenia, bipolar disorder, mania or hypomania or with a personal or family history of any neurological disorder were excluded from the study. Psychiatric comorbidities of patients included in the study are reported in Table S1. The final sample included 52 CA and 36 HC. Demographic data, history and current substance use were collected (see Table 1 for sample details).

**TABLE 1:** Demographics, brain size, impulsivity in study samples and current and past substance abuse

	Patients with cocaine addiction (*N* = 52)	Healthy controls (*N* = 36)	*χ* ^2^ or t	*p*
Age (M ± SD, years)	31.3 ± 6.51	30.1 ± 7.62	0.795	0.429
Education (M ± SD, years)	10.9 ± 2.9	13.2 ± 3.53	−3.276	0.002
Total intracranial volume (M ± SD, μl)	1442.7 ± 104.6	1460.4 ± 98.31	−0.796	0.428
BIS total score (M ± SD)	61.1 ± 14.6	40.2 ± 10.4	6.52	<0.001
BIS attentive score (M ± SD)	17.1 ± 5.23	11.6 ± 5.23	4.79	<0.001
BIS motor score (M ± SD)	18.4 ± 7.79	13.3 ± 5.72	2.97	0.004
BIS nonplanning score (M ± SD)	25.6 ± 6.82	15.3 ± 5.29	6.70	<0.001
Duration of cocaine use (M ± SD, years)	10.8 ± 6.4			
Age of onset of cocaine use (M ± SD, years)	20.7 ± 4.99			
Mean dose of cocaine (grams) per week in the last year (dose = *n*)	0.33 = 6 0.33–0.66 = 11 1–4 = 22 4–8 = 5 8–10 = 2 >10 = 3 n/a = 3			
Method of drug administration (*n*)	Smoking = 35 Inhalation = 13 Both = 4			
Time since last use (M ± SD, days)	11.6 ± 10.4			
Polysubstance abuse				
Tobacco (*n*)	43			
Duration of tobacco use (M ± SD, years)	14.6 ± 8.06			
Benzodiazepines (*n*)	2			
Cannabis (*n*)	4			

Abbreviations: BIS, Barratt Impulsiveness Scale; M, mean; SD, standard deviation.

Moreover, the Barratt Impulsivity Scale (BIS‐11), which allows the measurement of impulsivity and its subdomains, including attentional, motor and non‐planning impulsiveness, was administered.^23^ All participants underwent a detailed cognitive assessment of cognitive flexibility, inhibition, working memory, decision‐making and executive functions using the following tests^29^: Berg's card sorting test (BCST), flanker task, go/no‐go task, letter number sequencing, digit span backward, Iowa gambling task and Tower of London. Cognitive performance was compared between CA and HC using analysis of covariance (ANCOVA) with age and education as a nuisance variable.

### 2.2 Imaging acquisition

Brain images were acquired on a Philips Ingenia 3T MR system with a 32‐channel head coil with the following parameters: T1‐weighted images were acquired using a three‐dimensional FFE SENSE sequence, repetition time/echo time (TR/TE) = 7/3.5 ms, field of view = 240, matrix = 240 × 240 mm, 180 slices, gap = 0, plane = sagittal, voxel = 1 × 1 × 1 mm (five participants were acquired with a voxel size = 0.75 × 0.75 × 1 mm) and scan time = 3.19 min.

### 2.3 Preprocessing

We used the Statistical Parametric Mapping analysis package (SPM12) together with the Computational Anatomy Toolbox for SPM (CAT12). For preprocessing and analysis, we applied default parameters in accordance with a standard protocol (http://www.neuro.uni-jena.de/cat12/CAT12-Manual.pdf).

In detail, T1 images were spatially registered to the Montreal Neurological Institute (MNI) template using diffeomorphic anatoical registration through exponentiated lie algebra (DARTEL) registration. Brain structural data were segmented into GM, white matter (WM) and cerebrospinal fluid (CSF) and then used for the reconstruction of the cortical surface for each participant using the projection‐based thickness method.^30^ The total intracranial volume (TIV) was calculated as the sum of the volumes of GM, WM and CSF. The central surface reconstruction included topology correction, spherical inflation and registration. The central surface was used as input to calculate the CCF values. Finally, all surface measures for both hemispheres were merged and resampled at a resolution of 32 k mesh. We also included a two‐step quality check: First, all images were visually inspected for artefacts before preprocessing. Then, after segmentation, the images underwent a statistical quality control for inter‐subject homogeneity and image quality, as included in the CAT12 toolbox.

The CCF was analysed following the specifics implemented in CAT12, using the ‘spherical harmonic reconstruction’ approach proposed by Yotter and colleagues.^16^ To increase the signal‐to‐noise ratio, given the average distance between the sulci and the gyri, the resampled surface data for the CCF were smoothed using a 25‐mm (and repeated using a 20 mm to exclude a significant effect of the smoothing filter) Gaussian full width at half maximum (FWHM) kernel before the second level analyses.

### 2.4 Statistical analysis

Demographic data were compared between the CA and HC groups using two‐sample *t*‐tests. The total and subdomain scores of BIS‐11 were compared between the groups using an ANCOVA, with age and years of education (for BIS‐11 scores) as covariates. A voxel‐wise general linear model with age as a covariate was used to compare the CCF between the two groups. Nonparametric permutation‐based testing was applied to t‐stat maps using the threshold‐free cluster enhancement (TFCE) method with 10 000 permutations to correct for multiple comparisons with the family‐wise error (FWE) approach at the cluster level with α = 0.05. To exclude that drug administration methods could have affected our results, CA was stratified for this variable, and CCF was compared using one‐way ANOVA. Furthermore, to rule out a possible confounding effect of education differences between groups, ANCOVAs were performed on the CCF values of the clusters showing a significant effect of diagnosis with years of education as a nuisance covariate. Furthermore, to investigate the correlation between clinical, cognitive and imaging data in CA, we used a partial Pearson's correlation between addiction duration indexes (duration/age of onset), weekly dose (average weekly dose in grams of cocaine in the last year), BIS total and subscale scores, cognitive performance on each neuropsychological test and CCF in those clusters showing an effect of diagnosis using education as a covariate.

## 3 RESULTS

### 3.1 Demographic and clinical data

The groups did not differ with respect to age (CA, 31.28 ± 6.51 years; HC, 30.08 ± 7.62 years; *p* = 0.428) and showed a significant difference in education (CA 10.94 ± 2.90 years, HC 13.19 ± 3.52 years, *p* = 0.001). The mean age of onset of CA was 20.65 ± 4.99 years. The drug administration methods were smoking only (*n* = 35), inhaling only (*n* = 13) and both (*n* = 4). Current polysubstance use included cannabis (*n* = 4) and benzodiazepines (*n* = 2). None of the patients were using opioids or amphetamines at the time of evaluation (Table 1). CA patients had a higher BIS‐11 total (*p* < 0.001) in all subdomain scores: attentional (*p* < 0.001), motor (*p* = 0.004) and non‐planning (*p* < 0.001) when compared with HC (Table 1). Regarding cognitive performance, CA showed poorer performance compared with HC at the BCST in the following indexes: categories completed (*p* < 0.001, *t* = −4.14); categories experienced (*p* < 0.001, *t* = −4.14); correct responses (*p* = 0.008, *t* = −3.37); and total mistakes (*p* = 0.008, *t* = 3.41). All other cognitive assessments did not show significant differences between the diagnostic groups.

### 3.2 CCF

**FIGURE 1:**
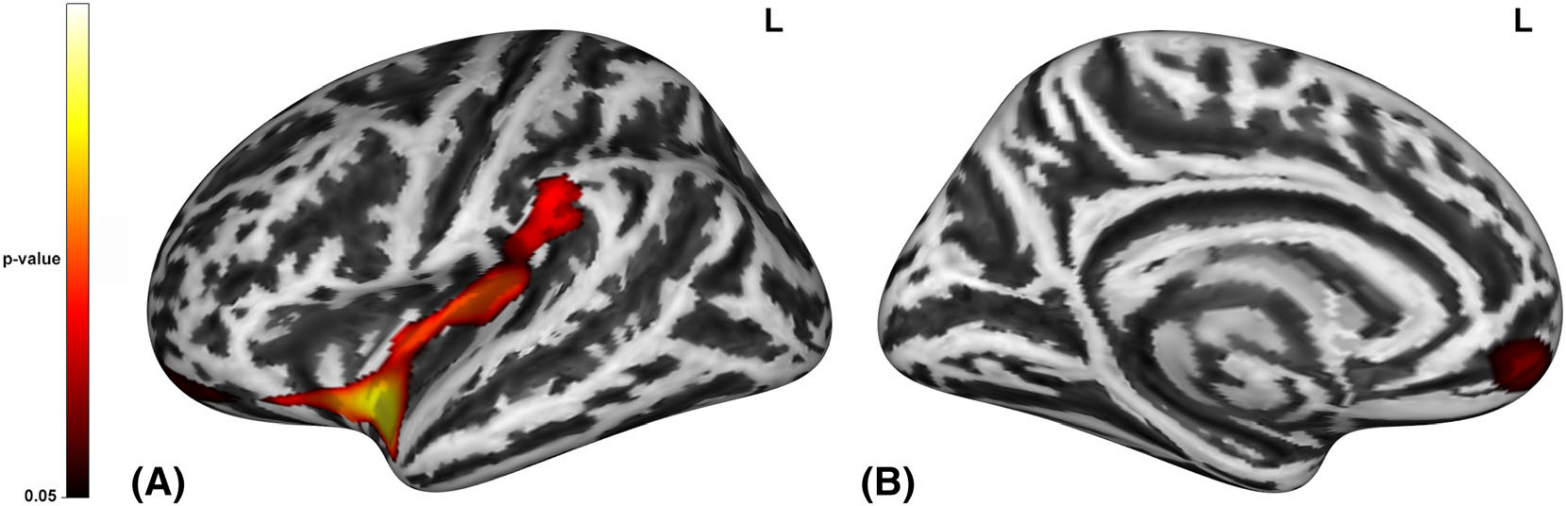
Increased complexity of cortical folding (CCF) in patients with cocaine addiction (CA) in the left (A) lateral and (B) medial hemispheres. CCF was reduced in patients with CA in a cluster that spans the left insula and the left part of the supramarginal gyrus (A) and in the left medial orbitofrontal cortex (B) compared with healthy controls (HCs). Statistical maps are displayed at *p* < 0.001 uncorrected and *p* < 0.05 family‐wise error (FWE) cluster‐level corrected. The colour bar represents the *p*‐value.

CA showed a reduced CCF compared with HC in a cluster that included the left insula and the left part of the supramarginal gyrus (SMG, cluster peak at x, y, z = −37, 7, −10; k = 1162, *p* = 0.008) (Figure 1A) and in the left medial OFC (mOFC, cluster peak at x, y, z = −27, 48, 8; k = 307, *p* = 0.039) (Figure 1B). There were no significant differences in CCF within the CA group between the type of drug administration in the left insula and the left SMG cluster [F (2, 49) = 0.243, *p* = 0.784] and the left medial OFC cluster [F (2, 49) = 0.477, *p* = 0.623]. These results did not change when the 20‐mm Gaussian FWHM kernel smoothing was applied and was confirmed by ANCOVAs with education as a nuisance covariate.

### 3.3 Correlations with cocaine use characteristics, impulsivity scores and cognitive performance

**FIGURE 2:**
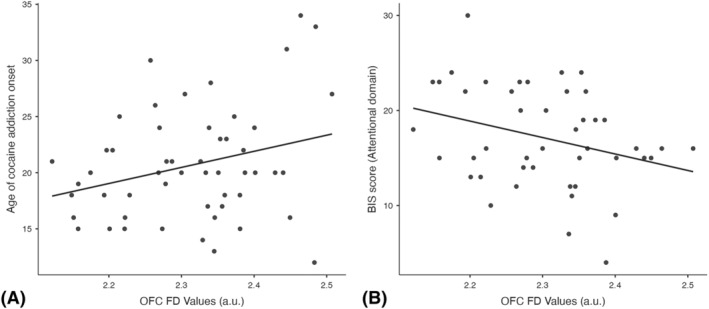
Scatter plot of the complexity of cortical folding (CCF) and the age of onset of cocaine addiction (A) and the attentional subdomain of the Barratt Impulsiveness Scale (BIS) (B). The CCF in the left medial orbitofrontal cortex (OFC) was positively correlated with the age of onset of cocaine addiction (A). The CCF in the left medial orbitofrontal cortex (OFC) was negatively correlated with the attentional subdomain of the BIS (B). Age is measured in years, CCF in arbitrary units (a.u.) and BIS score is an absolute value. The line represents the best fit. FD, fractal dimension

CCF values in the medial OFC were positively correlated with the age of onset of CA (*r* = 0.310, *p* = 0.028) (Figure 2A). In addition, in the CA group, the CCF values in the medial OFC were negatively correlated with the attentional subdomain of the BIS score (*r* = −0.307, *p* = 0.048) (Figure 2B). No other correlations were found between CCF values and cocaine dose or impulsivity scores. Finally, we did not find any significant correlation with cognitive performance.

## 4 DISCUSSION

To our knowledge, this is the first study to investigate changes in cortical complexity in patients with chronic CA. Our main finding is that patients with chronic CA, compared with controls, showed a lower CCF in the left insula, supramarginal gyrus and medial OFC. The CCF in the left medial OFC was positively correlated with the age of onset of CA and negatively with the total years of CA. Furthermore, CCF in the left medial OFC of cocaine users was negatively correlated with the attentional subdomain score of the BIS scale.

A wide body of research has shown that drug taking behaviours that occur after exposure to substances may be related to altered neural circuits involved in motivation, decision‐making and learned associations.^31^ More specifically, the prefrontal cortex (PFC), and in particular the OFC, appears to be a key player in the development of addictions due to its role in decision‐making, reward‐based and goal directed‐behaviour.^32^ The PFC is essential for cognitive processes such as attention, working memory, decision‐making, cognitive control and delay discounting, all of which are compromised in addicted individuals.^33^ Clinical studies have reported a pattern of generalized PFC dysfunction in drug‐addicted individuals, which appears to be associated with worse outcomes (e.g., increased drug use, poor performance of PFC‐related tasks and higher probability of relapse).^33^ Additionally, structural imaging studies have shown a reduction in PFC CT in individuals with SUD, not only for cocaine but also for other substances.^34^ Within the PFC, GM loss is more evident in dorsolateral PFC, ACC and OFC and is correlated with longer duration and greater severity of drug use.^34^ Cortical thinning in the OFC and in the insula has been previously reported in CA and is associated with long‐lasting changes in the OFC that affect voluntary control. This may be due to a general decrease in baseline metabolic activity in this region and a reduction in dopamine D2 signalling.^35^ Additionally, the disruption of OFC has been associated with compulsive behaviour and disinhibition.^3^ We found a decrease in CCF in the left mOFC in CA. Reduced CCF has been associated with altered brain structure in neurodegenerative disorders characterized by cognitive impairment, including Alzheimer's disease, frontotemporal dementia and mild cognitive impairment.^36^ Reduced CCF can be associated with impaired cognitive performance and may underlie the reduced response inhibition ability that is associated with impulsivity (vide infra). In particular, we found a correlation between CCF in the left mOFC and the age of onset of cocaine use, suggesting a dose‐effect relationship between cocaine use and the organization of brain structure. Notably, this result is in line with a recent longitudinal investigation showing that changes in CT in the frontal cortex in cocaine users were related to the amount of cocaine consumed during the study period.^11^

**FIGURE 3:**
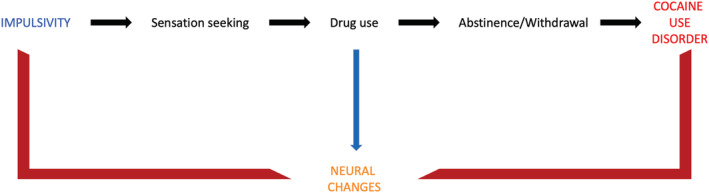
Relationship between neural changes, impulsivity and cocaine use disorder. Neural changes can cause increased impulsivity and cocaine use disorder (CUD), which, together with the direct effects of the substance, also have neuroplastic effects on the brain that increase the risk of impulsivity and CUD, thus perpetuating the disorder with a feedforward mechanism. On the psychopathological level, impulsivity directly and through increased sensation seeking (that can also be considered a dimension of impulsivity) can cause risk behaviours, including substance abuse. After discontinuation or decrease in substance consumption, withdrawal and abstinence symptoms will appear, ultimately resulting in a cocaine use disorder.

Furthermore, the cluster of reduced CCF in OFC was negatively correlated with the attentional domain of the BIS. Consistent with the literature on cocaine use, patients with CA had greater impulsivity that affected all subdomains in our study.^37^ Furthermore, CA is characterized by impairments in attentional skills and a significant attentional bias toward cocaine‐related stimuli.^38^ In general, patients with SUD have a general impairment in the processing of reward‐related stimuli due to cognitive and attentional biases.^21^ Our results suggest that impaired impulsivity and ultimately cognitive biases in SUD may be related to the reduced CCF of the OFC^21^ (Figure 3). Similar findings of a correlation between GM volume and addictive behaviour have been described in animal models. Alterations in cortical and sub‐cortical GM volume have been correlated with behavioural sub‐dimensions of addiction, such as high motivation for drug taking (medial PFC), maintenance of drug use despite negative consequences (periaqueductal grey) and persistence of drug seeking (motor, somatosensory, association, insular cortices and amygdala).^39^ Animal studies investigating the impact of chronic use of cocaine on drug‐related behaviour and brain structure in rats have found that cocaine exposure can induce persistent structural alterations in the regions implicated in addiction, such as nucleus accumbens, ventral pallidum, striatum, substantia nigra, insular cortex and OFC.^40^ Furthermore, these changes appear to be most pronounced when drug exposition occurs early during adolescence. These findings suggest that cocaine use could induce brain changes that contribute to the reinforcement of addicted behaviour.^41^ The impairment of set‐shifting abilities in our study supports this idea.^42^

We also found a reduction in CCF in the left insula in CA. Insula is involved in the integration of visceral sensations, which can affect the decision‐making process in addiction.^10^ This region sends input to the OFC where they can inform decisions and guide actions.^43^ In particular, the connections between the insula and the ventro‐tegmental area, and the substantia nigra, can play a crucial role in the development of addictions with modulation of dopamine signalling.^43^ Animal models investigating the learning processes underlying the association of external signals with the rewarding effects of drugs have shown that the insula is involved in the perception of bodily needs that guide motivated behaviours with a key role for the interoceptive insular cortex in drug craving in animals exposed to amphetamine.^44^ Recent investigations have found significant alterations in the insular cortex in patients with different types of addiction,^45^ including cannabis, online gaming, social media and smoking. In particular, a reduction in GM volume and CT in the insula was demonstrated in patients with cocaine and heroin addiction.^5^ Structural alterations in the insula may affect the interaction between cognitive and affective processes in decision‐making and ultimately contribute to the lack of avoidance responses to aversive events that can underlie drug‐seeking behaviour and craving.

We found reduced CCF in SMG. Recent studies have indeed shown that a reduction in GM volume in SMG predicts craving symptoms in CA.^46^ Reduced connectivity in a brain circuit between SMG and the ventral striatum that is involved in emotional perception and awareness, has been associated with stimulant addiction.^47^ Thus, the role of altered cortical complexity of SMG may be associated with an altered ability to control impulsive responses, including craving, motivational effects and the maintenance of addicted behaviour through cognitive control deficits such as dysregulation of incentive salience assigned to drug‐related stimuli.^48^

Overall, our study provides evidence that cocaine abuse alters the CCF in specific brain regions involved in interoception, decision‐making and response inhibition. With our cross‐sectional design, we cannot determine whether our findings predispose to or result from addiction. However, the relationship between altered CCF in OFC and the duration of cocaine abuse suggests that this alteration may follow local neurotoxic or neuroplastic effects.^49^ This observation is consistent with evidence from animal studies that shows that repeated exposure to cocaine can induce long‐lasting changes in brain morphology, including inhibition of neurite extension, reduction in endoplasmatic reticulum dilation, abnormal lysosomal proteolysis and altered neuronal mitochondrial dynamics.^50^ Notably, these cocaine‐dependent cellular and molecular adaptations have been associated with changes in the dynamics of gene expression mediated by epigenetic changes, including DNA methylation, histone modifications and microRNAs.^51^ At the same time, although impulsivity is considered both a determinant and a consequence of drug use, including cocaine, our findings of a relationship between the reduced CCF in this region and attentional impulsivity argue in favour of a preexisting condition that can underlie the risk of the disorder. CA is a complex disorder with genetic and environmental factors that play an important role individually and in interaction,^49^ and the neurobiology revealed by CCF appears to support this idea.

On the other hand, previous studies have investigated the relationship between CT, gyrification, cortical surface area and CCF (which are thought to have distinct neurodevelopmental trajectories) and impulsivity traits in healthy young adults.^52^ Overall impulsivity was associated with a higher local gyrification index (LGI), particularly in temporo‐parietal regions, with separate regions predicting distinct types of impulsivity: fronto‐temporo‐parietal regions for nonplanning impulsivity and fronto‐parietal and occipital areas for attentional impulsivity.^52^ The authors suggested that variations in LGI (a marker of early neurodevelopment that was altered in the fronto‐temporo‐parietal cortex) could lead to increased impulsivity in healthy individuals. Furthermore, CT (but not surface area) in the temporal, superior parietal and occipital cortex was negatively associated with higher global impulsivity in healthy individuals.^53^ Taken together, these findings suggest that alterations in brain structure and in cortical folding, in particular, may reflect abnormalities in neurodevelopment and are associated with impulsivity traits also in healthy subjects without exposure to substances, thus representing a possible signature of vulnerability to addiction behaviours that predates the neuroplastic effects of substances. These theories are also supported by previous findings of cortical alterations in individuals with other SUD (i.e., cannabis, alcohol, hallucinogens and stimulants) and addictive behaviours (i.e., online gaming), suggesting that cortical abnormalities might be involved in the pathophysiology of addictions. Moreover, recent evidence has shown that cortical surface changes are associated with impulsivity in neurodegenerative disorders,^12^ supporting the theory that structural cortical abnormalities could contribute to increased impulsivity, which in turn represents a risk factor for SUD.

Overall, the relationship between cocaine abuse and cortical alterations is complex and not yet fully clarified. Not only can CCF alterations be a sign of a vulnerability trait leading to cocaine use disorder (CUD) (through impulsivity traits) but the substance itself can also cause changes in the cerebral cortex. Although genetic factors can directly contribute to cocaine dependence (heritability = 0.4–0.7),^54^ cocaine itself can affect gene expression in the PFC and the midbrain, leading to functional and structural changes in the brain, including synaptic plasticity and neural connectivity, which are partially stable and can contribute to addiction and relapse in CUD.^54^ Moreover, animal studies show that prolonged exposure to cocaine can lead to impaired attention and this bias is selectively mediated by altered OFC activity.^55^

We must acknowledge some limitations of this study. First, the sample consisted only of men, which limits the generalizability of the results to women. However, addiction in general and cocaine presents several differences between the sexes, including the severity of craving, medical and psychiatric comorbidity and social, family and employment problems.^28^ Therefore, including only men can have reduced the heterogeneity of our results. Second, our study is cross‐sectional and therefore cannot determine the causal relationship between brain changes and SUD. Despite these limitations, to our knowledge, this is the first study to investigate the CCF in patients with CA.

In conclusion, we show that cortical surface morphometry, measured by the CCF, is altered in CA. Our results support the idea that the development of CA may be associated with neurobiological alterations that underlie the vulnerability to this disorder. In its turn, the use of cocaine can affect the neural circuits that mediate behaviour that support the addiction process itself with a feedforward mechanism.

Future longitudinal studies with a larger sample size including subjects with other substance or behavioural addictions are warranted to unravel the contribution of these processes in the development of addictions.

## CONFLICT OF INTEREST

The authors declare no conflict of interest.

## AUTHOR CONTRIBUTIONS

*Conceptualization, methodology, data processing, statistical analysis, visualization, writing the original draft, reviewing and editing*: Nicolò Trevisan. *Writing the original draft, reviewing and editing*: Fabio Di Camillo. *Writing the original draft, reviewing and editing*: Niccolò Ghiotto. *Writing the original draft, reviewing and editing*: Giulia Cattarinussi. *Writing the original draft, reviewing and editing*: Maddalena Sala. *Conceptualization, reviewing and editing and project administration*: Fabio Sambataro.

## Supporting information


**Table S1.** Psychiatric comorbidities of patients.

## Data Availability

The data that support the findings of this study are openly available in OpenNeuro at doi:10.18112/openneuro.ds003346.v1.1.2, reference number ds003346.
