# Supplementary material for: The complexity of cortical folding is reduced in chronic cocaine users
Source: Addict Biol. 2023 Feb 1;28(3):e13268. doi: 10.1111/adb.13268 (PMC10078524; doi:10.1111/adb.13268)
Supplement: Supplementary file 1 — Table S1. Psychiatric comorbidities of patients. [file ADB-28-0-s001.docx]

**SUPPLEMENTARY MATERIAL**

**The complexity of cortical folding is reduced in chronic cocaine users**

Nicolò Trevisan^1,2^, Fabio Di Camillo^1^, Niccolò Ghiotto^1^, Giulia Cattarinussi^1,2^, Maddalena Sala^1^, Fabio Sambataro^1,2^

^1^ Department of Neuroscience (DNS), University of Padova, Padua, Italy

^2^ Padova Neuroscience Center, University of Padova, Padua, Italy

Corresponding author:

Fabio Sambataro

Department of Neuroscience (DNS), University of Padova, Padua, Italy

Via Giustiniani 5, Padova

[Tel:+390498211980](about:blank)

e-mail: [fabio.sambataro@unipd.it](mailto:fabio.sambataro@unipd.it)

**Table S.1 Psychiatric comorbidities of patients.**

| **Psychiatric Comorbidities** | **N^a^** |
| --- | --- |
| Major Depressive Episode current (2 weeks) | 5 |
| Major Depressive Episode Recurrent | 5 |
| Substance Induced Mood Disorder | 3 |
| Major Depressive Episode with Melancholy | 2 |
| Anxiety Disorder with panic due to a general medical condition current | 1 |
| Social Phobia current (past month) | 3 |
| Specific Phobia current | 3 |
| Generalized anxiety disorder current (past 6 months) | 10 |
| Substance induced generalized anxiety disorder current | 1 |
| Adjustment Disorders current | 1 |
| Post-traumatic Stress Disorder current (past month) | 3 |
| Somatization Disorder current | 1 |
| Hypochondriasis current | 1 |
| Conduct Disorder (past 12 months) | 1 |
| Attention Deficit/ Hyperactivity Disorder (adults; current) | 17 |
| Alcoholic dependence (past 12 months) | 22 |
| Substance dependence (non-alcohol; past 12 months) | 48 |
| Antisocial Personality Disorder | 17 |
| ^a^, data are available for 51 patients | |

**ANCOVA of CCF values with education years and BIS score as covariates.**

After performing an ANCOVA of the CCF values, with education years and BIS score as covariates, between healthy controls and patients with cocaine addiction, the difference between the two groups were still significant both in the cluster located in the Insula [F(2,68) = -4.15, p < 0.001] and the cluster in the orbitofrontal cortex [F(2,68) = -2.61, p = 0.011].
